# The Book World of Medicine and Science

**Published:** 1903-04-04

**Authors:** 


### The Book World of Medicine and Science.

Constipation. By G. Sherman Bigg, F.R.C.S.E. (London: Baillière, Tindall and Cox. 1902. 2s. 6d. net.)

We cannot avoid an uneasy suspicion that this book was written for the public rather than for the profession. We can hardly believe that Mr. Bigg would otherwise have thought it necessary to describe as he does in the language of an elementary teacher the various processes of digestion. "The food," he says, "is first masticated by the teeth into a pulp, so as to mix with the saliva, which changes the insoluble starch material into a soluble variety of sugar, and acts as a lubricant to facilitate the passage of the food down the gullet." "The digestion in the stomach is chiefly carried on by the action of the gastric juice, a secretion composed of pepsin, hydrochloric acid, water, and other salts. The pepsin is the active principle," etc., etc. We quote these sentences to show that the book is intended to be read by the populace, and we contrast the elementary instruction therein contained with the long series of prescriptions given at the end of the book. If the book is meant for the medical profession a good deal of it is unnecessary, while if it is meant for the public a very large portion of it is extremely dangerous, as being likely to lead to injurious self-drugging. Constipation, surely, of all diseases in the nosology, has been the most extensively maltreated by amateur attempts at cure, and anything which may tempt sufferers to physic themselves for the relief of this condition is much to be deprecated. Otherwise there is a good deal to be said for the book. It is full of hints and suggestions which, although not presenting any novelty, may possibly be useful to junior practitioners. We confess that we should like to know more about chionanthes virginica than we are told in the long but very unexplanatory paragraph devoted to the subject. We are informed in regard to an American preparation of this substance that "this is not, in any sense of the word, a secret or quack remedy, as any physician who cares to take the trouble can readily ascertain the nature of the ingredients; but if the nature of this compound is so easily ascertained why should not an author who so strongly recommends its use tell us something more about it, instead of girding at those jealous people who use the prescribing of this drug "as a stone to throw at a successful practitioner who avails himself of all the latest resources of pharmacy?" These are things that spoil a book which with a little care might have been made into a useful monograph.

The Thompson Yates Laboratories Report. Edited by Rupert Boyce and C. S. Sherrington. (London: Messrs. Longmans, Green and Co. 1902. Royal quarto 563 pages. Eight plates. 20s.)

This is a large volume, full of most interesting information. It stands to reason that the articles in a book of this kind appeal chiefly to specialists; in fact, it may be said that the published report of any great laboratory must be a book written by specialists for specialists. On the other hand, the subjects selected for publication in the present volume cover a very wide range; for neuro-pathology is represented by a paper on "The Injury Current of Nerve," by J. S. MacDonald, M.B.; bacteriology is the subject of several of the communications; and even pathological anatomy finds a place in the shape of an excellent description of a post-mortem on a case of multiple aneurisms of the aorta by Dr. Hill Abram and Dr. Lyn Dimond. Perhaps the most curious subject for investigation was that selected by Dr. H. E. Annett, who caused the sputum expectorated on to the sidewalks of the streets of Liverpool to be collected and taken to his laboratory, where he examined them for tubercule bacilli, and tested their capability of producing tuberculosis in the lower animals. Rather more than one hundred of these "crachats" were collected and examined, and it was found that three of them contained tubercule bacilli, and two others produced tuberculous lesions when inoculated, so that a little less than 5 per cent. were undoubtedly capable of carrying infection. As drying does not affect the virulence of tubercule bacilli, and as the amount of sunlight which reaches the pavement in the large towns of this country is probably small, the expectoration of phthisical patients in the streets must be considered a distinct source of danger to the public. The last paper in the volume is a report, by Dr. H. E. Durham, of the mission to Parà, which was undertaken by Dr. Myers and himself for the purpose of investigating the etiology of yellow fever, and a melancholy interest attaches to it in consequence of the death of Dr. Myers while engaged in the work. This mission was under the auspices of the Liverpool School of Tropical Medicine and Medical Parasitology. The report includes a discussion on the claim of Sanarelli's bacillus icteroides to be considered the parasite of yellow fever, and Dr. Durham sums up against such a claim. Reference is also made to the work done by the Americans on the same subject. The author deals with the various theories of the etiology of the disease, and he is convinced that the view that a parasite is injected into the body by the bite of a gnat is the correct one. This view is strongly supported by the fact that the disease is not infectious or contagious in the ordinary way, but that infection clings to certain houses in a locality, these houses being, of course, infested by gnats which have sucked the blood of a yellow-fever patient, and therefore contain the poison. Further, the bites of gnats which had fed on yellow-fever patients were found by the Americans to give the disease to human beings. It is thought that the virus incubates in the gnat for from 12 to 20 days, and that the gnat may remain infectious for 57 days. Dr. Durham was able to isolate, from the organs and stools of yellow-fever patients, a fine bacillus which presented certain distinctive features, but as he was not successful in cultivating it outside of the body, he has not been able to prove that it is the actual germ of the disease.

